# The secrets of calcicole species revealed

**DOI:** 10.1093/jxb/eraa555

**Published:** 2021-02-24

**Authors:** Alexander Lux, Jana Kohanová, Philip J White

**Affiliations:** 1 Department of Plant Physiology, Faculty of Natural Sciences, Comenius University in Bratislava, Bratislava, Slovakia; 2 Institute of Chemistry, Slovak Academy of Sciences, Bratislava, Slovakia; 3 Ecological Sciences Department, The James Hutton Institute, Invergowrie, UK

**Keywords:** Apoplasmic barriers, calcicole species, calcium, element toxicity, endodermis

## Abstract

This article comments on:

**Kotula L, Clode PL, Ranathunge K, Lambers H**. 2021. Role of roots in adaptation of soil-indifferent Proteaceae to calcareous soils in south-western Australia. Journal of Experimental Botany **72**, 1490–1505.

**Calcium (Ca) is an essential macronutrient for plants. It serves as a structural component of cell walls and membranes, contributes to charge balance and osmotic regulation, and participates in cellular and systemic signalling (White and Holland, 2018). However, excess Ca can be toxic. In this issue, Kotula *et al.* (2021) have investigated the adaptive mechanisms enabling survival of soil-indifferent Proteaceae on soils with high Ca availability. To do this, they compared pairs of congeneric species, one of which was calcifuge and the other soil indifferent, in the genera *Hakea* and *Banksia*. They observed that the mechanisms of adaptation to excess Ca included both theoretical possibilities: (i) restricted Ca uptake and translocation in the soil-indifferent *Hakea* by apoplasmic barriers in the root; and (ii) tolerance of large tissue Ca concentrations by sequestration in specific cell types in the shoot in the soil-indifferent *Banksia*.**

## The control of calcium fluxes across roots

Ions move across the root to the vascular tissues either extracellularly (apoplasmic pathway) or, following their uptake by root cells, through their interconnected cytoplasm (symplasmic pathway). Both transport pathways have advantages and disadvantages. The symplasmic pathway allows flux control and specificity, whilst the apoplasmic pathway allows rapid movement of potentially toxic nutrients, such as Ca and Zn, to the xylem.

The development of the root endodermis largely determines the pathways and location of Ca movement across the root to the vascular tissues (White, 2001; Moore *et al.*, 2002). The apoplasmic movement of Ca across the root to the vascular tissues is restricted by the development of the endodermal Casparian strip (state I endodermis) and the impregnation of cell walls by suberin and/or lignin. The symplasmic movement of Ca to the vascular tissue, which is thought to occur primarily through the cytoplasm of endodermal cells, is then restricted by the suberization of endodermal cells (state II endodermis). Subsequently, the thickening of cell walls, accompanied by lignin deposition (state III endodermis), further restricts Ca fluxes to the vascular tissue. In some plant species this stage may be accompanied by silica deposition. Differences in endodermal ontogeny among Proteaceae, and their relationships to Ca transport to the shoot, are clearly shown by Kotula *et al.* (2021). In most angiosperm species, roots also develop an exodermis, with properties similar to the endodermis (Enstone *et al.*, 2002), but this was not observed in the Proteaceae studied by Kotula *et al.* (2021).

## Adaptations enabling the survival of soil-indifferent Proteaceae in environments with high Ca availability

Kotula *et al.* (2021) have focused on the role of roots in enabling soil-indifferent Proteaceae to survive environments with high Ca availability. They investigated the relationships between the accumulation of Ca in shoots, the development of the endodermis, and the cell-specific distribution of Ca in the roots. They found that the two soil-indifferent Proteaceae used two different strategies to avoid Ca toxicity.

Strategy I is adopted by the soil-indifferent species *Hakea prostrata*. When given a high Ca supply, the calcium non-tolerant *H. incrassata* accumulates much more Ca in its shoot than *H. prostrata*, which parallels transpiration, and suggests that *H. prostrata* restricts Ca and water movement across the root in these conditions. Greater suberization of the endodermis close to the root apex, restricting both apoplasmic transport and Ca influx to endodermal cells, is likely to account for the smaller Ca flux to the shoot of *H. prostrata*.

Strategy II is adopted by the soil-indifferent species *Banksia prionotes*. When given a high Ca supply, both Ca-non-tolerant (*B. menziesii*) and soil-indifferent (*B. prionotes*) *Banksia* have similar shoot Ca concentrations, and neither the development of the root endodermis nor Ca accumulation in root cells differs between these species. This suggests that restricting movement of Ca across the root does not contribute to calcicole behaviour in *B. prionotes*. It is likely that the ability of *B. prionotes* to grow in soils with high Ca availability is related to its tolerance of high tissue Ca concentrations and the ability of this species to sequester Ca in specific cell types in the shoot, thereby avoiding compromising P nutrition, which the calcifuge species *B. menziesii* cannot do (Hayes *et al.*, 2019).

## Exclusion and tissue tolerance are common adaptations of plants to excess toxic elements in the environment

Accelerated development of the root endodermis (strategy I) appears to be a general strategy to restrict the accumulation of potentially toxic elements in shoots. For example, it has been observed as an adaptation or acclimatory response restricting the uptake and translocation of potentially toxic elements in both saline and metalliferous environments (White *et al.*, 2001; Lux *et al.*, 2011; Barberon 2017). In particular, accelerated development of the endodermis confers greater tolerance to high concentrations of Na (Karahara *et al.*, 2004), Zn (Bokor *et al.*, 2014), Cd (Lux *et al.*, 2004, 2011), and Sb (Vaculíková *et al.*, 2016) in the rhizosphere. The enhancement of endodermal development, such as the development of peri-endodermal layers, can be observed in several species that hyperaccumulate transition metal elements (Broadley *et al.*, 2007). Intriguingly, the rate of development of the endodermis, as well as ectopic lignification or suberization that also restricts solute movement, are often sensitive to the local rhizosphere environment. For example, the development of suberin lamellae and premature (ectopic) development of apoplasmic barriers are observed locally in sections of roots exposed to excess toxic elements (Lux *et al.*, 2011; Líška *et al.*, 2016).

Another role of apoplasmic barriers is to restrict the ‘back-flux’ of nutrients from the vascular tissues of the root to peripheral tissues. Wang *et al.* (2019) recently reported that the loss of integrity of the Casparian strip in Arabidopsis mutants leads to solute leakage, but that this can be prevented by the deposition of suberin, which leads to the rebalancing of water and nutrient uptake.

The sequestration of potentially toxic elements in specific shoot cells (strategy II) appears to be a general strategy of plants to facilitate tissue tolerance and is observed in many plants that can tolerate high tissue concentrations of these elements (White and Pongrac, 2017). Classic examples of this phenomenon are the accumulation of Ni, Cu, Zn, and Pb in the epidermis of plants that hyperaccumulate these elements (White and Pongrac, 2017). In contrast, although most Cd is accumulated in the epidermis, large concentrations are often present throughout the leaf, and Mn is accumulated in the upper layer of palisade mesophyll cells or in the subepidermal cells of plants that hyperaccumulate these elements (White and Pongrac, 2017).

Another mechanism conferring tissue tolerance is the biomineralization of potentially toxic elements (He *et al.*, 2014). Elements sequestered in biominerals include C, transition metals, Ca, S, and P. Ca salts, such as calcium oxalate (CaC_2_O_4_), calcium sulfate (CaSO_4_), calcium carbonate (CaCO_3_), calcium phosphates, and calcium phytate (C_6_H_6_Ca_6_O_24_P_6_), can be precipitated in the apoplasmic space or vacuole to remove excess Ca and potentially toxic anions (Franceschi and Nakata, 2005; He *et al.*, 2014; White and Holland, 2018).

## Calcicole flora

Calcicole species are, by definition, associated with Ca-rich habitats, but not necessarily calcareous soils. Relatively few plant species are able to colonize calcareous soils, which are dominated by CaCO_3_, and the flora of calcareous soils is often determined by tolerance to P and Fe deficiencies (Lee, 1999). The flora of calcareous soils is considered to be a consequence of their evolution on base-rich, dry soils typical of a Mediterranean climate (Chytrý *et al.*, 2010). In contrast, the flora of gypsum soils appears to be related to the ability of plants to tolerate high Ca and S availability in the environment, through either exclusion or tissue tolerance (Mota *et al.*, 2016). The specific adaptations of calcicole species are often associated with their endemism (Gao *et al.*, 2020). The proportion of the calcicole flora that restrict Ca uptake and transport to the shoot or accumulate Ca in specific shoot tissues is currently unknown. However, it has been observed that the tissue nutritional Ca requirement and the minimal concentration of Ca producing Ca toxicity are often greater in calcicole species than in calcifuge species (White and Holland, 2018), and plants that grow on gypsum substrates often biomineralize CaSO_4_ (Mota *et al.*, 2016), which suggests that tolerance of high tissue Ca concentrations is critical for calcicole ecology. Nevertheless, as noted by Mota *et al.* (2016), there is a general lack of studies on the adaptation of plants to Ca-rich environments.

## Conclusion and perspective

Kotula *et al.* (2021) have revealed two strategies enabling soil-indifferent Proteaceae to survive environments with a high Ca supply. The first strategy is to restrict Ca uptake and translocation to the shoot by accelerated development of the root endodermis. The second strategy is to tolerate large tissue Ca concentrations by sequestration of Ca in non-photosynthetic cells to avoid compromising P nutrition. It is likely that these strategies are shared by other calcicole species, but there has been no comprehensive survey of adaptations in root anatomy or the partitioning of Ca among shoot cells in the calcicole flora. Perhaps now is the time to fill this knowledge gap with pertinent data.
